# Principal component analysis of photoplethysmography signals for improved gesture recognition

**DOI:** 10.3389/fnins.2022.1047070

**Published:** 2022-11-03

**Authors:** Yuwen Ruan, Xiang Chen, Xu Zhang, Xun Chen

**Affiliations:** School of Information Science and Technology, University of Science and Technology of China, Hefei, Anhui, China

**Keywords:** gesture recognition, KNN, PCA, PPG, SVM

## Abstract

In recent years, researchers have begun to introduce photoplethysmography (PPG) signal into the field of gesture recognition to achieve human-computer interaction on wearable device. Unlike the signals used for traditional neural interface such as electromyography (EMG) and electroencephalograph (EEG), PPG signals are readily available in current commercial wearable devices, which makes it possible to realize practical gesture-based human-computer interaction applications. In the process of gesture execution, the signal collected by PPG sensor usually contains a lot of noise irrelevant to gesture pattern and not conducive to gesture recognition. Toward improving gesture recognition performance based on PPG signals, the main contribution of this study is that it explores the feasibility of using principal component analysis (PCA) decomposition algorithm to separate gesture pattern-related signals from noise, and then proposes a PPG signal processing scheme based on normalization and reconstruction of principal components. For 14 wrist and finger-related gestures, PPG data of three wavelengths of light (green, red, and infrared) are collected from 14 subjects in four motion states (sitting, walking, jogging, and running). The gesture recognition is carried out with Support Vector Machine (SVM) classifier and K-Nearest Neighbor (KNN) classifier. The experimental results verify that PCA decomposition can effectively separate gesture-pattern-related signals from irrelevant noise, and the proposed PCA-based PPG processing scheme can improve the average accuracies of gesture recognition by 2.35∼9.19%. In particular, the improvement is found to be more evident for finger-related (improved by 6.25∼12.13%) than wrist-related gestures (improved by 1.93∼5.25%). This study provides a novel idea for implementing high-precision PPG gesture recognition technology.

## Introduction

Photoplethysmography (PPG) sensors embedded in wearable devices currently on the market are often used for health detection (Raj et al., 2021; Dhar et al., 2022; Loh et al., 2022), identity verification (Alotaiby et al., 2021; Dae et al., 2021), and emotion recognition (Lee et al., 2019; Goshvarpour and Goshvarpour, 2020). In recent years, researchers have begun to explore the feasibility of using PPG signal for gesture recognition to achieve human-computer interaction. Taking advantage of PPG signals, Zhao et al. (2021) recognized 9 gestures with the average accuracy of 88%, and Yu et al. (2018) recognized 10 gestures with the average accuracy of 90.55%. Based on these studies, there is a growing consensus that PPG has the potential to replace EMG, accelerometer and other inertial sensors in the field of gesture recognition. Subramanian et al. (2020) conducted a comparative gesture recognition study on PPG and surface electromyography (EMG), and verified that the performance of these two types of signals was at the same level. Ling et al. (2021) proved that PPG signal is more suitable than acceleration signal for gesture interactions in wearable devices from three perspectives: (1) PPG is less affected by background motion noise; (2) PPG has better recognition performance of finger-related gestures; (3) PPG is more robust in reducing training burden. However, although PPG gesture recognition technology has made some progress, it is still in early research stage. The recognition accuracy and robustness have not met the needs of commercial use yet.

The main principle of using PPG signals for gesture recognition is that hand movement can cause deformation of blood vessels or tissues, resulting in PPG signal change with different movement patterns. In occasions such as heart rate estimation or blood oxygen detection, it is necessary to reduce motion artifacts to prevent their influence on measurement performance. However, for human-computer interaction, the motion information contained in the PPG signal is the key to realize gesture recognition, on the contrary, the vital sign-related information is regarded as noise. Consequently, the traditional PPG signal motion artifacts elimination methods are no longer applicable in this case.

When gesture actions are used for human-computer interaction, the signals collected by PPG sensors contain components related to multiple factors such as heart rate, gesture relevant motion and gesture irrelevant motion. In terms of signal sources, the components can be regarded as independent of each other, and the component irrelevant to gesture motion is not conducive to gesture recognition. Meanwhile, the energy levels of PPG signals caused by different factors may vary greatly. When there exist high-energy noise components, gesture recognition accuracy is bound to suffer significantly. Based on above analysis, we believe that if the PPG signals can be decomposed into components corresponding to different factors, it is expected to improve the accuracy of PPG-based gesture recognition through effective noise reduction.

In aspect of signal decomposition algorithms, principal component analysis (PCA), independent component analysis (ICA), and empirical mode decomposition (EMD), etc., can realize the effective decomposition of multi-channel or single-channel signals, and have been widely used in processing of biomedical signals such as electroencephalograph (EEG) (Turnip and Junaidi, 2014; Agarwal and Zubair, 2021; Farsi et al., 2021) and EMG (Masri et al., 2021; Xiong et al., 2021) etc. In PPG-based measurement of heart rate and other vital sign parameters, PCA, ICA, and EMD also have been adopted to remove motion artifacts, and the ability of these signal decomposition algorithms in separating the components related to vital signs and motion artifacts have been confirmed. For instance, Lee et al. (2020) proposed an ICA-based motion artifact reduction algorithm for PPG heart rate measurement. Liu et al. (2021) presented a PCA-based scheme to remove motion artifacts in PPG signals for blood pressure measurement. Adopting a method based on ensemble EMD and PCA, Motin et al. (2018) realized the estimation of heart rate, respiratory rate, and respiratory activity from PPG signals. For pulse rate detection, Wang et al. (2010) proposed an approach based on EMD and Hilbert transform to reduce the artifacts in PPG signals.

Inspired by the research progress of motion artifacts elimination based on signal decomposition algorithms, this study tried to explore the feasibility of using signal decomposition technology for separating gesture-related signals from physiological noise and gesture irrelevant motion noise, and subsequently developed effective PPG processing scheme for improving the accuracy of PPG gesture recognition. Its main innovation and contribution lie in that: (1) PCA algorithm was applied to decompose multi-channel PPG signals associated with 14 kinds of gestures, and the characteristic analysis of different principal components was carried out to explore the feasibility of separating gesture pattern related signal from irrelevant noise; (2) An effective gesture PPG signal processing method based on normalization and reconstruction of principal components was accordingly developed and carried out.

## Materials and methods

### Gesture set and data acquisition

**FIGURE 1:**
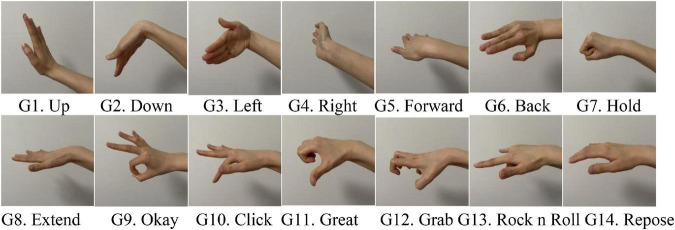
Fourteen gestures in the target gesture set.

In this study, the target PPG gesture dataset is the same as that in our previous work (Ling et al., 2021). As shown in Figure 1, the target gesture set consists of 14 kinds of wrist and finger joint related actions. Gestures G1–G6 mainly focus on wrist movements, gestures G7–G13 focus on finger movements, and gesture G14 is a baseline gesture, which requires the hand in a natural relaxed state. The execution process of each gesture starts from the resting state that the arm is naturally drooping and relaxed. When performing the gestures, subject raises the arm in front of the chest, completes the action, and finally returns to the relaxed state.

Fourteen healthy subjects (8 males and 6 females) participated in the data acquisition. The subjects were between 22 and 24 years old. All of them are right-handed and have no known history of any neural or musculoskeletal disease. All of the subjects were informed about the experiments and signed an informed consent form (No. PJ 2014-08-04) approved by the Ethics Review Committee of First Affiliated Hospital of Anhui Medical University. Each subject was asked to finish a set of experiments in four exercise states, namely: Sitting, Walking (speed of 3 km/h), Jogging (speed of 5 km/h), and Running (speed of 8 km/h). Except Sitting, the experiments in the other three exercise states were carried out on a treadmill (Sole F63) to make sure that the subjects exercise at a constant speed. In the states of Sitting and Walking, the subjects were asked to repeat each gesture about 50 times, while in the states of Jogging and Running, each gesture was repeated about 25 times. There was a 2-s interval between repetitions, and subjects were asked to rest about 5 min between gestures to avoid muscle fatigue.

**FIGURE 2:**
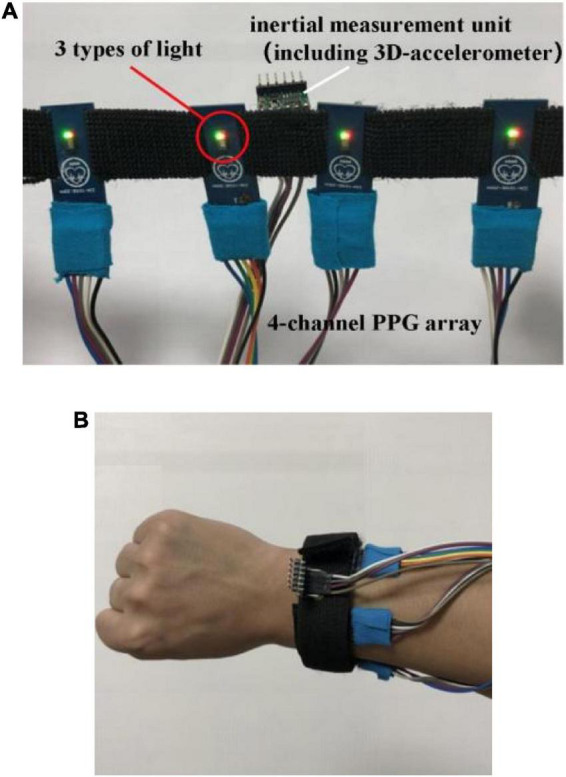
Schematic diagram of **(A)** the acquisition device and **(B)** the wearing example.

A wristband-type multi-channel PPG acquisition device (Figure 2A) developed and manufactured by the research team was used for data collection. The device was equipped with four PPG sensors (MAX30105, Maxim Integrated Inc., San Jose, CA, USA). As shown in Figure 2B, the device was worn on the right forearm at distance of about one finger from the wrist and were placed near radial artery, ulnar artery, cephalic vein and guillotine vein, respectively, to obtain PPG signals from the four major blood vessels. Each PPG sensor alternately emitted the red light, infrared light and green light, and the 4-channel signals of the same light from the four PPG sensors were collected synchronously. The sampling frequency of each channel was set to 100 Hz.

### Principal component characteristic analysis of gesture photoplethysmography signals

The PCA algorithm is adopted to decompose multi-dimensional data into a series of linearly uncorrelated elements called principal components using orthogonal transformation (Bro and Smilde, 2014; Lever et al., 2017). As depicted in Equation 1, the gesture PPG signal matrix *X*(4-channel, *N* data points) is transformed into principal component matrix *PC* and the corresponding weight vector matrix *W* using PCA algorithm according to the specific steps as follows:


$$PC^{4\times N}=W^{4\times 4}X^{4\times N}$$

(1) $$X={\left[X_{1},X_{2},X_{3},X_{4}\right]}^{T},W={\left[W_{1},W_{2},W_{3},W_{4}\right]}^{T}$$

$$PC={\left[PC1,PC2,PC3,PC4\right]}^{T}$$

(1). Perform de-averaging operation on each row of *X*, which is also called zero-averaging;
(2). Calculate the 4 × 4 covariance matrix according to Equation 2;

(2) $$\sigma =\frac{1}{N-1}XX^{T}$$

(3). Perform eigenvalue decomposition on the covariance matrix to find the eigenvalues and corresponding eigenvectors, and arrange the eigenvectors in descending order of eigenvalues to get the eigenvector matrix *W*;
(4). Calculate the principal components according to Equation 3.

(3) PCi=ωiTX

Then the characteristic analysis of different principal components is carried out to explore the feasibility of separating gesture pattern related signal from irrelevant noise. Principal component characteristic analysis is carried out in time domain and frequency domain, respectively. In the time domain, the envelope, energy, and variance levels of each channel of the raw PPG signals and each principal component are analyzed. In the frequency domain, Fast Fourier Transform (FFT) is performed on the raw PPG signals and principal components, respectively, to obtain the corresponding frequency domain signals. The frequency distributions of each channel of the raw PPG signals and each principal component are analyzed through mean frequency (*MNF*) and median frequency (*MDF*) calculated by Equations 4, 5, respectively, where *M* is the number of the frequency points, *f_j_* is the frequency of point*j* and *P_j_* is its power spectrum.


(4) $$MNF=\frac{\sum_{j=1}^{M}f_{j}P_{j}}{\sum_{j=1}^{M}P_{j}}$$

(5) $$\sum_{j=1}^{MDF}P_{j}=\sum_{j=MDF}^{M}P_{j}=\frac{1}{2}\sum_{j=1}^{M}P_{j}$$

In order to depict the relationship between the principal components and the raw PPG signals, Pearson correlation coefficient (Ly et al., 2018) is used to measure the correlation between the signal sequences.

### The photoplethysmography gesture recognition scheme based on principal component analysis processing

**FIGURE 3:**
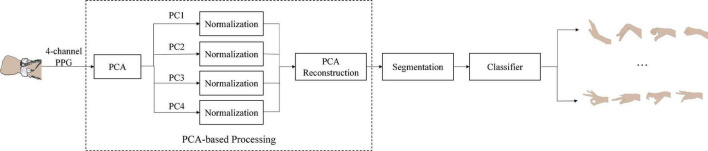
The PPG gesture recognition scheme based on PCA processing.

Figure 3 shows the PPG gesture recognition scheme based on the proposed PCA processing method, including: collecting 4-channel gesture PPG signals, PCA processing of the 4-channel PPG gesture signals, segmentation of gesture action activity; and gesture recognition with SVM classifier and KNN classifier. Gesture PPG signal collection is the same as described above, and details of the other steps are described below.

The purpose of PCA processing is to reduce the negative impact of high-energy noise components on gesture recognition by normalizing different principal components to the same level, so as to achieve the effect of denoising. For the 4-channel gesture PPG signals, the detailed procedure of the PCA processing is as follows:

(1). Decompose the 4-channel PPG signals into four principal components by means of PCA algorithm as described above.
(2). Normalize the four principal components with unequal energy to the same level. In particular, for each *PC*, to find the maximum and minimum values firstly, then normalize the data points greater than zero to [0 1] by the maximum value, and normalize the data points less than zero to [−1 0] by the absolute value of the minimum;
(3). Obtain the processed PPG signals by reconstructing the normalized principal components according to Equation 6.

(6) $$X_{processed}=W\cdot PC_{normalized}$$

The goal of gesture segmentation is to extract gesture repetition samples from continuous signals. A motion background noise-based segmentation strategy proposed in our previous work (Ling et al., 2021) is adopted in this study. In this strategy, considering that the waveform and amplitude of gesture signals are far greater than the motion background signal, the starting and ending positions of a gesture repetition is determined by setting suitable thresholds. The details of the motion noise-based strategy can be found in Ling et al. (2021). In addition, the lengths of the gesture repetitions are not always consistent in different exercise states in this study. In the states of Sitting and Walking, the completion time of a gesture is about 1 s. As the movement speed increasing, the time to complete a gesture will get shorter, and the gesture completion time in the Running state is about 0.5 s. According to the sampling rate of 100 Hz, the signal length of a gesture repetition is about 40–120 data points. To normalize the length of gesture samples, the down-sampling method is used to extract a 32-point envelope of each gesture repetition as gesture feature sample in further classification. Thus, each PPG gesture sample is in the form of a matrix of size 4 (channels) ×32 (data points).

Considering the limited number of samples in the target database, two classical and traditional machine learning classifiers, namely Support Vector Machine (SVM) classifier and K-Nearest Neighbor (KNN) classifier are adopted for gesture classification in this study. SVM is considered as one of the most successful machine learning algorithms in recent years (Cortes and Vapnik, 1995). The basic idea is to solve a separation hyperplane that can correctly classify the training data set, and the hyperplane needs to satisfy the maximum geometric interval between dissimilar data. SVM can solve the nonlinear case by choosing a suitable kernel function, using nonlinear processing to map the samples to a high-dimensional space, and then finding the best classification hyperplane in the high-dimensional space. In our previous work (Ling et al., 2021), the SVM classifier achieve the best performance in PPG gesture recognition compared to Convolutional Neural Network (CNN) and Long Short-Term Memory (LSTM). KNN algorithm originally proposed by Cover and Hart (1967). Since it is easy to realize and needs less training time, KNN classifier is commonly used in the field of pattern recognition. After the analysis of the distribution characteristics of gesture PPG samples and the experimental verification, for SVM classifier, a linear kernel function is used, and the penalty factor is set to 1. And for KNN classifier, the Euclidean distance is selected, and K is set to 1.

In view of the large individual differences in PPG signals, as a primary study, PPG gesture recognition in this study is carried out in a subject-specific way. In the meanwhile, considering that it is difficult to obtain large-scale gesture training data in practical human-computer interaction applications, this study focuses on gesture recognition with small training size. In particular, for each gesture, all samples of each subject are randomly divided into 10 parts, one part of samples is used for training data, and the remaining nine parts testing data. That is, 10% data is used for training the classifier and 90% for testing. The result of the recognition task is obtained by cross-validation method.

### Two denoising schemes for comparison

In order to evaluate the effectiveness and superiority of the proposed PCA processing method, gesture recognition adopting two common signal denoising methods, namely Butterworth low-pass filter (Li, 2007; Liao et al., 2014) and wavelet threshold denoising (Pan et al., 2007; Cheng and Zhang, 2014; Wang et al., 2019), have also been conducted in this study. Because the main spectrum of PPG gesture signals is concentrated in 0∼5 Hz, a 5-order Butterworth low-pass filter with a cut-off frequency of 5 Hz is adopted.

The wavelet threshold denoising is carried out as follows: (1) Calculate the orthogonal wavelet transform of the noisy PPG signal, decompose the signal into 4 layers and get the corresponding wavelet decomposition coefficient. The wavelet used is “sym6”; (2) Threshold the wavelet coefficients to obtain the estimate of the wavelet coefficients of the real signal. In specific, the soft threshold function shown in Equation 7 is adopted, where *th* is the threshold and γ is the wavelet coefficient. The minimax thresholding, which is defined as Equations 8, 9, is used to determine the threshold, where *N* is the sum of the total number of wavelet coefficients of the noisy signal on scales 1 4, *J* is the binary scale, and *W*_*1,k*_ is the wavelet coefficients of scale 1; (3) Do the inverse wavelet transform, and regard the reconstructed signal as the de-noised signal.


(7) $$T\left(\gamma ,th\right)=\left\{ \begin{array}{c} sgn\left(\gamma \right)\left(\left|\gamma \right|-th\right),\left|\gamma \right|\geq th\\ 0,\left|\gamma \right|<th \end{array}\right.$$

(8) $$th=\left\{ \begin{array}{c} \sigma \left(0.3936+0.1892log_{2}N\right),N>32\\ 0,N<32 \end{array}\right.$$

(9) $$\sigma =middle\left(|W_{1,k}|,0\leq k\leq 2^{J-1}-1\right)/0.6745$$

### Statistical analysis

Considering the data in this study do not strictly satisfy the conditions of normal distribution and homogeneity of variance, non-parametric tests (Kruskal–Wallis test) were performed to explore the impacts of the independent variables (wavelength, motion state, denoising method, and classifier) on the dependent variable (recognition accuracy). The statistical analysis was carried out on IBM SPSS Statistics (Version 25), and the significance level is 5%.

## Results and analyses

### Principal component characteristics analysis of multi-channel gestural photoplethysmography signals

**FIGURE 4:**
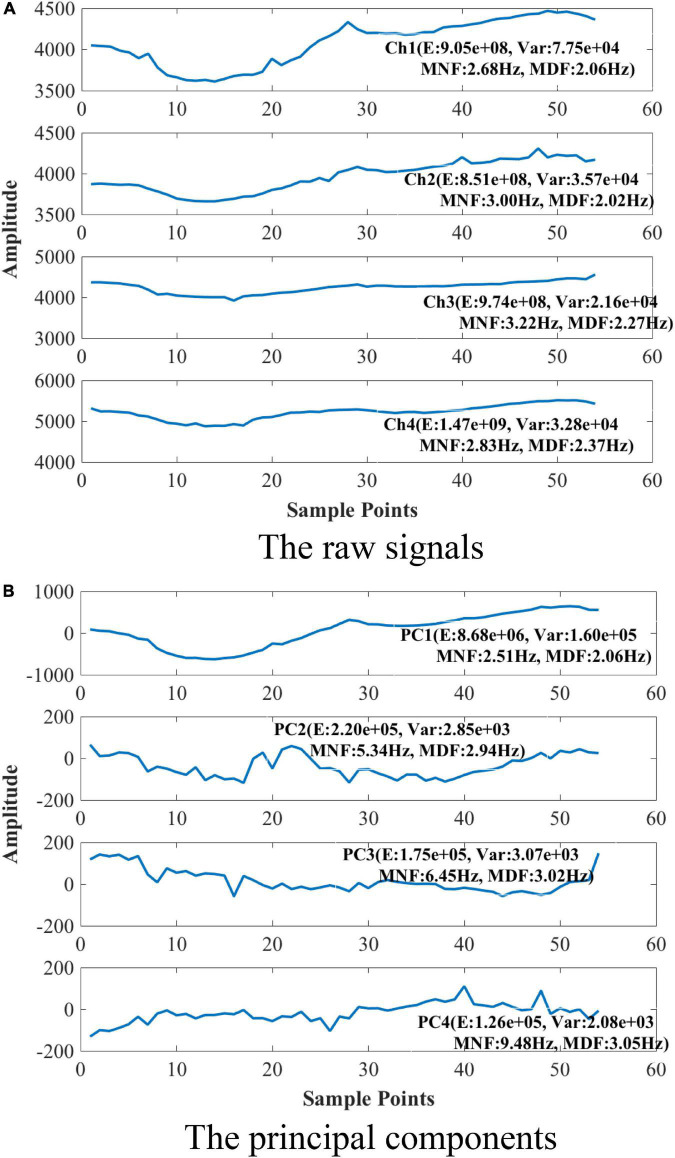
Raw signals and PCA decomposition results of a green light PPG sample (PC, principal component; E, energy; Var, variance; MNF, mean frequency; MDF, median frequency). **(A)** The raw signals. **(B)** The principal components.

Figure 4 shows the raw signals and PCA decomposition results of one PPG sample of gesture G5 from a representative subject (Sub11) in the Running state. From Figure 4A, it can be seen that the raw signals of different channels have similar changing trend (average similarity coefficient of pairwise channels ρ 0.92). The four channels are at almost the same level in terms of energy. The *MNF* and *MDF* of the four channels only vary slightly. In contrast, as shown in Figure 4B, the envelopes of the 4 principal components obtained by PCA decomposition are more distinct (average similarity coefficient of pairwise components ρ 0.35). For example, *PC*1 has the highest energy (8.68e + 06), which is more than 39 times higher than the other three components (1.26e + 05∼2.20e + 05). At the same time, the four components have significant frequency differences. *PC*1 has the lowest *MNF* (2.51 Hz) and *MDF* (2.06 Hz), while *PC*4 has the largest *MNF* (9.48 Hz) and *MDF* (3.05 Hz). Above results demonstrate that PCA can effectively decompose multi-channel gesture PPG signals into multiple components with different energy levels and frequency bands.

**FIGURE 5:**
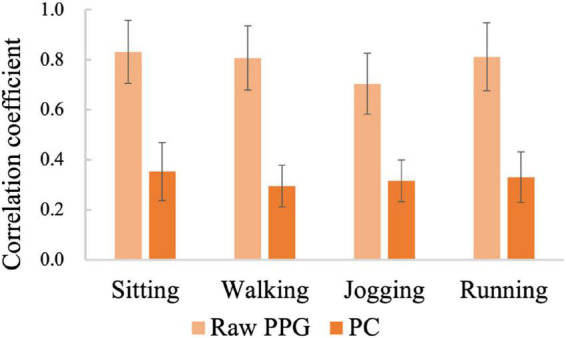
The similarities between the four channels of raw PPG signals and between the four principal components (Subject 11).

To explore which principal components are beneficial to gesture recognition, all gesture samples of the subject in the four motion states were analyzed. Figure 5 shows a similarity comparison between the four channels of raw PPG signals and the four principal components for all gesture samples, by calculating the correlation coefficients of pairwise channels or pairwise *PC* components (mean ± standard deviation). In all the 4 motion states, the average correlation coefficients of raw PPG are up to 0.7, while those of the principal components are only in the range of 0.29 and 0.35. These results verify that the four channels of raw signals have a large common component, however, the four principal components are much independent of each other.

**FIGURE 6:**
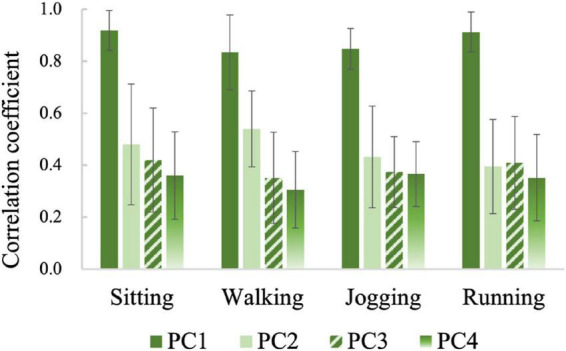
The similarities between principal components and raw PPG signals.

Figure 6 shows the similarities between the four principal components and the raw PPG signals for all gesture samples. The values (mean ± standard deviation) in the figure were obtained by calculating the correlation coefficients between one *PC* component and the four channels of raw signals and averaging them. From Figure 6 we can find that *PC*1 is similar to the raw PPG (ρ 0.83∼0.92) while *PC*2, *PC*3, and *PC*4 are less similar to the raw PPG (ρ 0.30∼0.54). According to the above analysis, we believe that component *PC*1, which has the largest energy level and the lowest frequency, maybe represents the common trend of the four channels of raw PPG signal.

**FIGURE 7:**
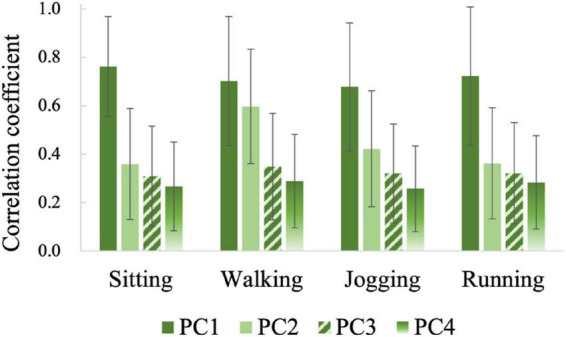
The similarities between the same principal components of the samples belonging to different gestures.

Figure 7 compares the correlation coefficients between the same principal components of the samples belonging to different gesture types. For samples belonging to different gestures, the mean correlation coefficients of *PC*1 are all up to 0.68, while those of *PC*2, *PC*3, and *PC*4 are mostly lower than 0.4. These results demonstrate further that *PC*1 mainly represents a common part in all kinds of gesture samples. This kind of trend item at a high energy level will mask the information related to gesture pattern, which may reduce the recognition accuracy. On the contrary, the other three principal components may contain more valuable gesture pattern related details.

In summary, PCA decomposition and principal component characteristics analysis of multi-channel gestural PPG signals verify the feasibility of using PCA to separate gesture pattern related signals from irrelevant motion noise.

### The photoplethysmography gesture recognition based on principal component analysis processing

#### Feature distribution of gesture samples obtained by different denoising schemes

**FIGURE 8:**
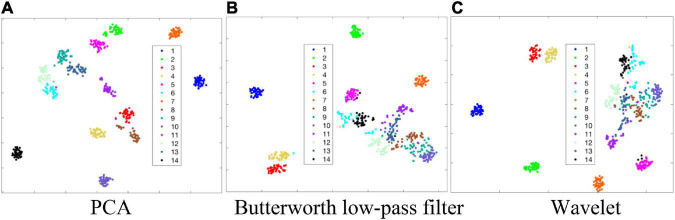
t-SNE dimensionality reduction feature distributions of gesture signal envelope in the cases of three denoising schemes. **(A)** PCA. **(B)** Butterworth low-pass filter. **(C)** Wavelet.

Taking the green light PPG samples of the 14 gestures of the 2nd subject (Sub 2) in the Sitting state as example, we perform t-SNE (van der Maaten and Hinton, 2008; Pezzotti et al., 2017) dimensionality reduction on PPG envelopes obtained by PCA processing, Butterworth low-pass filter and Wavelet threshold denoising, respectively. The obtained scatter plots of feature distribution are shown in Figure 8. In the cases of Butterworth low-pass filter and Wavelet threshold denoising, as shown in Figures 8B,C, respectively, there are six gestures that can be easily distinguished from other gestures. For the remaining eight gestures, the features are too close to meet the recognition condition. However, when PCA processing is applied, as shown in Figure 8A, the features of all 14 gestures can be separated effectively from each other. Above results demonstrate, to some extent, the possibility of improving gesture recognition accuracy by the proposed PCA processing scheme.

#### Recognition results for 14 gestures

Photoplethysmography gesture recognitions of 14 gestures are carried out in the cases of three lights and four motion states, using SVM and KNN classifiers, respectively. For each case, the average gesture recognition accuracies among 14 gestures and 14 subjects are shown in Table 1. In order to compare the effect of different denoising schemes and classifiers on PPG gesture recognition in the cases of different lights and different motion states, nonparametric tests are performed further and the results are shown in Table 2. Combined the Tables 1, 2, the following phenomena can be observed (The values presented in following parts, if not otherwise specified, are the average recognition accuracies and standard deviations for all recognition tasks involving the discussing factors):

**TABLE 1:** Hand gesture recognition accuracies (%) of 14 gestures.

Classifier		SVM			KNN		
Motion states	Lights	PCA	Butterworth	Wavelet	PCA	Butterworth	Wavelet
Sitting	Red	99.44 ± 0.41	93.57 ± 3.18	93.86 ± 3.17	99.25 ± 0.56	91.89 ± 4.43	92.16 ± 4.30
	Infrared	98.24 ± 1.65	94.02 ± 2.92	94.02 ± 3.16	97.70 ± 1.93	92.57 ± 3.79	92.41 ± 4.07
	Green	93.21 ± 5.20	88.38 ± 4.34	88.51 ± 4.50	91.22 ± 6.75	84.01 ± 6.62	84.14 ± 6.62
Walking	Red	98.81 ± 1.20	93.76 ± 4.12	93.96 ± 4.08	98.62 ± 1.47	92.04 ± 5.24	92.23 ± 5.11
	Infrared	97.87 ± 1.60	95.14 ± 2.37	95.28 ± 2.39	97.55 ± 1.51	93.97 ± 2.49	93.99 ± 2.82
	Green	92.86 ± 4.08	88.39 ± 6.35	88.55 ± 6.07	90.42 ± 5.35	84.70 ± 8.29	84.83 ± 7.97
Jogging	Red	95.43 ± 4.20	90.69 ± 5.97	91.59 ± 5.36	94.80 ± 4.38	89.51 ± 6.50	90.28 ± 5.97
	Infrared	95.31 ± 2.55	91.96 ± 5.60	92.96 ± 4.98	94.68 ± 2.97	91.35 ± 6.19	92.18 ± 5.66
	Green	89.57 ± 5.51	85.08 ± 8.60	85.47 ± 8.11	88.28 ± 5.59	83.48 ± 9.05	83.75 ± 8.60
Running	Red	97.00 ± 2.31	88.15 ± 4.22	88.74 ± 4.66	96.72 ± 2.35	87.53 ± 4.64	88.08 ± 5.14
	Infrared	94.19 ± 3.18	88.88 ± 3.89	89.22 ± 3.65	93.61 ± 3.33	88.15 ± 4.15	88.31 ± 4.06
	Green	88.48 ± 5.89	82.82 ± 8.51	83.82 ± 7.54	87.47 ± 6.94	81.30 ± 9.62	82.40 ± 8.25
Total		95.03 ± 4.86	90.07 ± 6.35	90.50 ± 6.05	94.19 ± 5.58	88.38 ± 7.28	88.73 ± 6.97

**TABLE 2:** The results of non-parametric tests for gesture recognition.

Factors		Sig. for accuracy	Multiple comparisons (Motion state)		Sig. for accuracy
Main	Light	0.000**	Running	Jogging	0.001*
	Motion state	0.000**		Walking	0.000**
	Processing method	0.000**		Sitting	0.000**
	Classifier	0.002*	Jogging	Walking	0.000**
				Sitting	0.000**
			Walking	Sitting	0.501

**Multiple comparisons** **(Light)**		**Sig.** **for accuracy**	**Multiple comparisons (Processing method)**		**Sig.** **for accuracy**

Green	Red	0.000**	PCA	Butterworth	0.000**
	Infrared	0.000**		Wavelet	0.000**
Red	Infrared	0.975	Butterworth	Wavelet	0.536

*p < 0.05, **p < 0.001.

(1). The wavelength of PPG signal has a significant impact on the recognition accuracy (*p* = 0.000^**^). Specifically, the recognition accuracy of green light (86.71 ± 7.53%) is significantly (*p* = 0.000^**^) lower than those of red light (93.25 ± 5.44%) and infrared light (93.48 ± 4.49%), and there is no significant difference between red light and infrared light (*p* = 0.975). From this result, it can be seen that although the green PPG suitable for heart rate detection is generally embedded in wearables, it is not the best choice for gesture recognition.
(2). The intensity of the motion has a significant impact on the recognition accuracy (*p* = 0.000^**^). In general, the recognition accuracy decreases with the increase of background motion speed: 92.70 ± 5.93% for Sitting, 92.94 ± 6.05% for Walking, 90.35 ± 7.01% for Jogging and 88.60 ± 6.92% for Running. However, there is no significant difference between Sitting and Walking (*p* = 0.501). This result shows that the low-intensity background motion has little influence on the recognition effect. Only movement that reaches a certain intensity makes the recognition accuracy decrease.
(3). The performance of the PCA processing is significantly (*p* = 0.000^**^) better than the other two denoising schemes. For the four motion states, three lights of PPG and two classifiers, the average recognition accuracies obtained by the three different denoising schemes are respectively: 94.61 ± 5.24% for PCA, 89.22 ± 6.87% for Butterworth and 89.61 ± 6.58% for Wavelet. Furthermore, the performances of Butterworth denoising and Wavelet denoising have no significant difference (*p* = 0.536). This result verifies the superiority of the proposed PCA denoising scheme.
(4). According to the results shown in Table 1, the recognition accuracy of SVM classifier (91.87 ± 6.20%) is slightly higher than that of KNN (90.11 ± 8.91%), and the statistical analysis result also shows that the classifier truly has some impact on the accuracy (*p* = 0.002*). However, in terms of the accuracy requirement of human-computer interaction, a difference of only one percent is not enough to conclude that SVM is superior to KNN. Considering the advantage of KNN in computing speed, it can still be considered that both SVM and KNN classifiers are well-suited for the PPG gesture recognition.

#### Recognition results for wrist-related gestures and finger-related gestures

**FIGURE 9:**
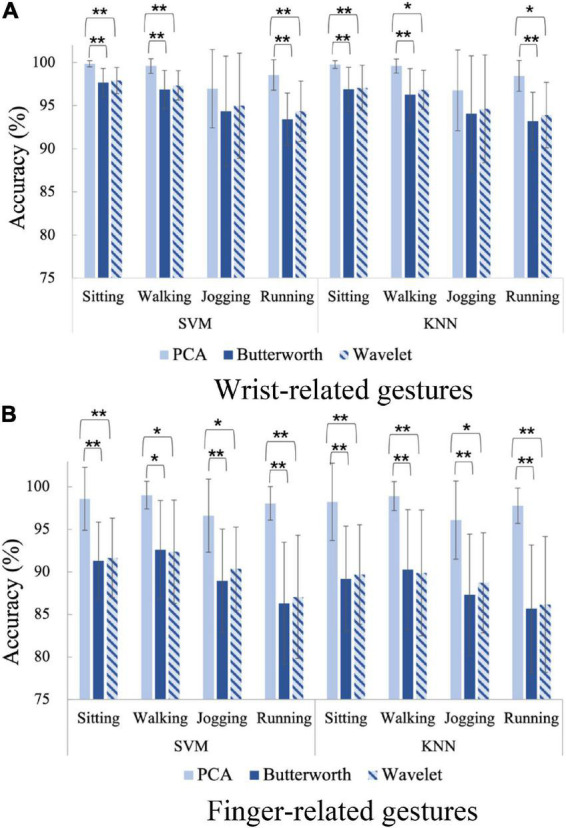
The gesture recognition results for wrist-related gestures and finger-related gestures under the four motion states, *p < 0.05, ^**^p < 0.001. **(A)** Wrist-related gestures. **(B)** Finger-related gestures.

As mentioned above, the target gesture set contains 6 wrist-related gestures (G1–G6) and 7 finger-related gestures (G7–G13). In order to compare the performance of the three denoising schemes on these two types of gesture, we further conduct recognition experiments on wrist-related gestures and finger-related gestures, respectively. The gesture recognitions are carried out using the red-light PPG data under the four motion states. Figures 9A,B give the recognition results of wrist-related gestures and finger-related gestures using SVM and KNN classifiers, respectively. Nonparametric tests are performed to explore the effects of denoising methods on recognition.

The recognition of finger-related gestures has always been a difficulty in the field of gesture recognition. As shown in Figure 9, when PPG gesture signals are denoised by general denoising means, the recognition accuracies of finger-related gestures are obviously lower than those of wrist-related gestures: 95.34 ± 4.23% for wrist-related and 88.95 ± 6.69% for finger-related when performing Butterworth low-pass filter denoising; 95.88 ± 4.02% for wrist-related and 89.49 ± 6.46% for finger-related when performing wavelet threshold denoising. In comparison, the proposed PCA denoising scheme can effectively improve the recognition performance of finger-related gestures. Applying the PCA processing scheme, the recognition accuracy of the finger-related gestures (97.91 ± 3.36%) is close to that of the wrist-related gestures (98.68 ± 2.70%). In the meanwhile, PCA processing improves finger-related gestured recognition accuracy better than wrist-related gestures. Taking the state of Running as example, for finger-related gestures, the average accuracy of PCA denoising scheme is 11.77% higher than Butterworth and 11.00% higher than Wavelet when using SVM classifier, and 12.13% higher than Butterworth and 11.63% higher than Wavelet when using KNN classifier. However, for wrist-related gestures, the average accuracy of PCA denoising scheme is only 5.14% higher than Butterworth and 4.21% higher than Wavelet when using SVM classifier, and 5.25% higher than Butterworth and 4.52% higher than Wavelet when using KNN classifier.

## Discussion

To the best of our knowledge, this is the first research paper that specifically focuses on gestural PPG signal denoising. Combined with the state of the art relevant to PPG gesture recognition technology, the research results obtained in this study can be discussed from the following aspects.

### The feasibility of separating gesture pattern related signals from irrelevant noises using principal component analysis algorithm

For PPG gesture recognition, how to extract gesture motion information from irrelevant motion and physiological signals is the key to determine the recognition performance. Inspired by the research progress of motion artifact elimination of PPG signal based on signal decomposition algorithm, this study carries out multi-channel PPG signal decomposition and principal component feature analysis using PCA algorithm to explore the feasibility of separating gesture pattern related signals from irrelevant noises. The experimental results demonstrate that PCA algorithm can effectively decompose the four-channel gesture PPG signals into four components with different energy levels and frequency bands. The component *PC*1, which has the largest energy level and the lowest frequency, maybe represent the common trend item of the four channels of raw PPG signal. We speculate that this kind of trend item at a high energy level will mask the information related to gesture pattern, thus reduce the recognition accuracy. On the contrary, the other three principal components may contain more valuable gesture pattern related details. The results of PCA decomposition and principal component analysis of gesture PPG signal provide a new way to propose effective signal processing method.

In fact, PCA algorithm has been widely used to decompose PPG signals to extract the needed physiological information. Liu et al. (2021) used PCA to separate the arterial pulse, capillary pulse and motion artifacts. Their acquisition device contains four colors of light, namely: blue, green, yellow, and infrared light, and one channel for each light. Because lights of different wavelengths reach the skin at different depths, the PPG signals of different lights can reflect different physiological information. In their study, based on PCA decomposition of the 4-channel PPG, *PC*1 was considered as arterial pulse which should have the largest energy. However, when PCA was applied to blue PPG and green PPG, *PC*1 was considered to be capillary pulse and *PC*2 was motion artifact. In the study of Motin et al. (2018) which used PCA with ensemble empirical mode decomposition (EEMD) to decompose PPG signals, the *PC*1 and *PC*2 were considered to represent heart and respiratory activity, respectively.

In summary, in related studies of physiological signal extraction, the first and second principal components which always have the largest energy, are often considered as the approximation of the required signals. However, in this study, we find that the first *PC* with the largest energy is likely the noise that interferes gesture recognition, and the last three principal components with low energy are more likely to be the gesture-pattern-related signal.

### The superiority of the proposed principal component analysis processing scheme in photoplethysmography gesture recognition

Although a number of studies have verified the feasibility of using PPG signals for gesture recognition in recent years, the problem of signal denoising has not attracted enough attention. In this study, a PPG processing method based on principal component normalization and reconstruction is proposed and implemented. The results of gesture recognition experiments on the data of 14 gestures, three kinds of light (red, infrared, and green) and four motion states (sitting, walking, jogging, and running) demonstrate the superiority of the PCA processing scheme from the following aspects.

First, the PCA processing method can improve the accuracy of gesture recognition to a certain extent (as shown in Tables 1, 2). As shown in Table 3, which summarizes representative studies in the field of PPG gesture recognition in recent years, most studies adopted common denoising methods such as Discrete Wavelet Transform (DWT) (Yu et al., 2018), Butterworth filter (Ling et al., 2021; Zhao et al., 2021), de-averaging (Subramanian et al., 2020), etc., for PPG signal processing. As we know, the objective of the common denoising methods usually is to remove motion noise that is not in the frequency band of gesture-related signals. In this study, compared with Butterworth denoising and Wavelet denoising, PCA denoising shows obvious superiority in improving the accuracy of PPG gesture recognition. This result verifies that, the simple denoising methods of removing signal in certain frequency band cannot sufficiently remove noise and thus improve the accuracy of gesture recognition. On the contrary, PCA is a better choice for PPG gesture recognition. Therefore, we should deeply explore PPG signal denoising scheme based on PCA and other signal decomposition algorithms in the future.

**TABLE 3:** The representative researches of PPG gesture recognition.

Author	Task	Light	Motion state	Denoising method	Classifier	The proportion of training data	Recognition accuracy
Zhao et al.	Nine finger-level gestures	Green	Stationary body-motion scenarios	Butterworth high-pass filter (cut-off frequency: 2 Hz)	GBT, ResNet	50%	88%
Yu et al.	Ten wrist/finger-level gestures	Green	Stationary (Sitting, leaning, standing), walking, taking transportation, jogging	Discrete Wavelet Transform (DWT), gradient-based denoising scheme	SVM, random forest classifiers	90%	73.6∼90.55%
Subramanian K. et al.	Four wrist/finger-level gestures	Green	Stationary	De-averaging, low-pass filter	SVM, XG-boosted trees	66%	88%
Ling et al.	Fourteen wrist/finger-level gestures	Red, infrared, green	Sitting, walking, jogging, running	Butterworth low-pass filter (cut-off frequency: 5 Hz)	SVM, CNN, LSTM	80%	83.3∼95.4 (95.28% for 6 wrist-related, 89.30% for 7 finger-related)
This study	Fourteen wrist/finger-level gestures	Red, infrared, green	Sitting, walking, jogging, running	PCA processing scheme	SVM, KNN	10%	87.47∼99.44% (98.68% for 6 wrist-related, 97.91% for 7 finger-related)

Second, the PCA processing scheme is of great significance for the realization of human-computer interaction based on finger-related gestures. From the perspective of actual application, the finger-related gestures are more suitable for interactive scenarios because they usually have more clear and easy-to-understand meaning. However, limited by factors such as small motion range and low degree of muscle contraction, when using electromyography or acceleration signals for gesture recognition, the distinguishability of finger joint motion is usually lower than that of wrist joint motion. In this study, the experimental results indicate that, for PPG gesture recognition technology, the distinguishability of finger-related gestures is also lower than that of wrist-related gestures. However, the proposed PCA processing scheme can improve the finger-related gestures recognition performance significantly. The PCA processing scheme is able to increase the recognition accuracy of finger-related gestures to a level similar to that of wrist-related gestures. With only 10% of the samples for training, the average recognition accuracy of seven finger-related gestures can achieve 97.91%, which can basically meet the commercial needs.

### The advantage of the photoplethysmography gesture recognition framework implemented in this study

Compared with the first three studies (Yu et al., 2018; Subramanian et al., 2020; Zhao et al., 2021) in Table 3, a relatively large target gesture set consisting of six wrist-related gestures, seven finger gestures, and a baseline gesture is targeted in this study, and the advancement of the research results is reflected in the following aspects:

1) Gesture recognition performance of PPG signals with different wavelengths is explored. Most current commercial wearable products are embedded with green PPG sensors because green light has a greater absorption rate for oxyhemoglobin and deoxyhemoglobin, which makes it more suitable for detection of physiological information. However, the experimental results in this study show that green PPG is not the best choice for gesture recognition. Regardless of motion state and denoising method used, the recognition performance of green PPG is obviously lower than that of red and infrared lights;
2) The recognition performance of PPG signals in more motion scenarios is investigated. In the works of Subramanian et al. (2020) and Zhao et al. (2021), PPG gesture recognition were carried out only in stationary state and simple body-motion scenarios. The work of Yu et al. (2018) added some motion scenarios such as walking and jogging. In this study, the background motion is designed more purposefully. We designed a series of tasks with sequentially increasing background motion velocities from 0 to 8 km/h, and strictly controlled the motion velocity to be constant using a treadmill. The experimental results demonstrate the low-intensity motion backgrounds such as walking at a speed of 3 km/h have little impact on the recognition, while the high-intensity movements have certain influences;
3) The gesture recognition framework proposed in this study has low training burden, which makes it valuable in the application of human-computer interaction. As shown in Table 3, the data for training accounts for 50∼90% of the target dataset in the relevant works. Considering the needs of practical application, we only use 10% of the samples to train the classifier in this study, which means that for each gesture, no more than 5 samples are included in the training dataset. Although the training test ratio is as low as 1:9, it still achieves satisfactory gesture recognition accuracy. In sitting state, using SVM classifier, the recognition accuracy of red PPG achieves 99.44%, and even the green PPG, which is not good at gesture recognition, achieves 93.21%.

Compared with our previous work published in 2021 (Ling et al., 2021), this study follows the same design of the gesture set and recognition tasks, but changes the gesture recognition framework. The recognition accuracies obtained in this study are significantly higher than those of our previous study. Taking Sitting state and SVM classifier as example, when the train test ratio is 1:9, the recognition accuracies are improved by 16.84% for red PPG, 14.84% for infrared PPG and 24.51% for green PPG. We believe the main reason why this study achieves better gesture recognition performance lies in that it adopts the proposed PCA processing scheme, which aims to weaken the noise component and highlight the gesture pattern related signal that is beneficial for gesture recognition. In our previous work, the signal preprocessing employed Butterworth low-pass filtering with a cutoff frequency of 5 Hz and amplitude normalization for each channel. According to the experimental result of this study, the denoising effect of Butterworth low-pass filtering is lower than the PCA processing method. At the same time, amplitude normalization also does not necessarily have a positive effect on gesture recognition. Since the four-channel PPG signals are collected at different locations on the wrist, each channel mainly responds to blood flow changes in different vessels. When performing different gestures, there should exist differences in the blood flow at the corresponding locations of these four channels, which helps to increase the distinguishability of the gestures. However, the normalization process puts the amplitude of the four channels at the same level, which means that the important difference information between gestures is erased. Therefore, the normalization is not conducive to gesture recognition.

Furthermore, SVM achieves the best performance for PPG gesture recognition in the previous work. Therefore, this study still uses the SVM classifier to complete the gesture recognition task. Meanwhile, considering that it is easier to implement and requires less training time, the KNN classifier is also adopted in this study. The experimental results show that, although the accuracy is slightly lower than that of SVM, the KNN classifier also has good recognition performance when using the proposed PCA processing scheme.

### Limitations and future work

Although this study provides a thought to improve the performance of PPG gesture recognition, it still has some limitations. First of all, the performance of the PCA processing method proposed in this study is only verified offline, but not tested online. Therefore, when the proposed method is applied to short signal sequences in online situations, the performance may degrade; Second, this study only takes the PCA algorithm as an example to preliminarily prove that the signal decomposition algorithm can effectively separate gesture pattern related signal from irrelevant noise. Of course, signal decomposition algorithms such as ICA, EMD, etc., may have the same function, and may even achieve better performance than the proposed PCA processing scheme. Third, the PPG gesture recognition in this study only uses a single batch of data and does not consider the effects of sensor displacement and other factors on the recognition performance. To promote the practicality of PPG gesture recognition technology, issues such as sensor displacement caused by repeated wearing of the device will be the focus of our future work.

## Conclusion

In this study, PCA decomposition technique is introduced into the noise processing of gesture PPG signals. After verifying the feasibility of using the PCA algorithm to separate the gesture pattern-related signals and irrelevant noises, a PCA processing method based on normalization and reconstruction is proposed and implemented. The superiority of the PCA processing scheme for improving the gesture recognition accuracy is verified in the recognition tasks of 14 gestures from 14 subjects, three kinds of light and four motion states, using two classifiers. In addition, the proposed PCA processing scheme is found to be more effective in improving the recognition accuracy of finger-related gestures. The research of this paper contributes to the development of PPG gesture recognition technology.

## Data availability statement

The raw data supporting the conclusions of this article will be made available by the authors, without undue reservation.

## Ethics statement

The studies involving human participants were reviewed and approved by the Ethics Review Committee of First Affiliated Hospital of Anhui Medical University. The patients/participants provided their written informed consent to participate in this study.

## Author contributions

YR designed the research scheme, did the data acquisition, data analysis, and gesture recognition experiments, and wrote the manuscript. XiC directed the research and substantially revised the manuscript. XZ and XuC participated in the interpretation of the research results and manuscript revision. All authors approved the final version of the manuscript.
